# Potential Role of Mast Cells in Intervertebral Disc Ageing, Herniation Resolution, and Degeneration: Evidence and Lessons Learned from Studies of Mast Cells in Other Connective Tissues

**DOI:** 10.3390/ijms27062804

**Published:** 2026-03-19

**Authors:** David A. Hart

**Affiliations:** Department of Surgery, Faculty of Kinesiology and McCaig Institute for Bone & Joint Health, University of Calgary, Calgary, AB T2N 4N1, Canada; hartd@ucalgary.ca

**Keywords:** mast cells, intervertebral disc, nucleus pulposus, annulus fibrosus, disc degeneration, sciatica, mast cell stabilizers, ketotifen

## Abstract

In the body, mast cells are found in the circulation and located in tissues. These immune cells arise in the bone marrow and are often associated with conditions such as allergies and asthma. However, these cells also play roles in other inflammatory reactions, dysregulated wound healing and chronic conditions. Regarding their presence in tissues of the intervertebral disc (IVD), mast cells have been located in the normal nucleus pulposus, and reports indicate mast cell numbers are elevated in IVD degenerative conditions. As the integrity of the IVD is reported to decline with ageing as well as in sciatica and clinically defined degenerative conditions, targeting mast cell function may be a viable conservative treatment option for the ageing IVD in health and disease. This review discusses the possible involvement of mast cells in IVD health and disease, and the rationale for the use of mast cell stabilizers such as ketotifen as potential treatment options for conditions affecting IVD integrity. Such mast cell targeting treatments may be considered alone or in combination with other molecules such as specific proteinase inhibitors impacting proteinases known to be present in the affected tissues, such as MMP-3 and HTRA1. Thus, a multicomponent approach in such treatments may provide effectiveness in inhibiting progressive loss of IVD integrity and function in chronic degenerative conditions or adverse outcomes due to non-resorption of extruded nucleus pulposus in sciatica.

## 1. Introduction and Purpose of the Review

A majority of people will experience low back pain in their lifetime, with >40% experiencing sciatica in their lifetime [1,2], and many others experiencing low back pain [3] and intervertebral disc (IVD) degeneration (degenerative disc disease, DDD). While the majority of those with sciatica will have their condition resolve, a subset of patients will have persistent pain that does not resolve. For the latter subset of patients with a non-resolving disc herniation at lumbar disc level 4/5 (L4/5) or lumbar disc level 5/sacral 1 (L5/S1), surgery is often the option of choice.

Similarly, many individuals develop chronic lower back pain as they age due to degeneration of the lumbar discs [4,5]. Again, the discs very often affected are L4/5 and L5/S1. The conditions associated with degeneration are most often treated with pain medications, exercise and physiotherapy until they reach a stage requiring surgery. While IVD integrity can decline or be altered during the ageing process, not all ageing individuals develop DDD so apparently, there are unique features leading to overt DDD.

While genetics likely plays a role in the onset of some of these conditions [6,7,8], as well as age [4,5], sex [5,9], smoking [10] and co-morbidities such as diabetes [10], treatment options are limited, and some have been tried and fallen out of favour for a variety of reasons. For example, treatment with opioids can have side-effects and addiction potential [11,12].

Therefore, new approaches are needed to both prevent development and inhibit the progression of these conditions and better understanding of the disease processes is needed for the human conditions. This review will discuss the potential of targeting mast cells and their products to take the interventions available in new directions. The discussion will focus on both evidence regarding IVD, as well as lessons learned from studies of mast cells in other connective tissues that may also provide support for similar roles in IVD regulation.

## 2. The Intervertebral Disc

The IVD is a multicomponent tissue (nucleus pulposus, NP; annulus fibrosus, AF; cartilaginous endplate), which, in concert with bone, contributes to the spinal column comprising many individual discs in different regions (i.e., the cervical, thoracic and lumbar) and terminating at the lower end at the transition point with the pelvis and the L5/S1 joint. The IVDs in different regions have some different functions, but generally they protect the spinal cord and regulate bipedal movement and movement of the head. The discs are designed to sustain compression but are also subjected to mechanical shear and tension in some motions as discussed in [13]. In many respects, the IVD functions as an organ system not unlike other joints such as the knee [14,15,16].

Each IVD comprises an inner NP and an outer annulus fibrosis (AF). In early life, the nucleus pulposus has a somewhat amorphous structure and consists of many proteoglycans and collagen type II, while the annulus fibrosis has a lamellar structure and each lamella mainly comprises collagen type I, some minor collagens, elastin, and proteoglycans [17]. Some molecules such as PRG4 are reported to primarily reside in the outer AF, with the inner AF and the NP devoid of this molecule known for its lubricating activity [18,19] as well as other roles [20]. The NP is likely designed to accommodate compression, while the lamellar structure of the AF is designed to resist compression and directional stresses [21,22], not unlike the inner and outer regions of the menisci of the knee [23,24]. The NP are mostly devoid of neural and vascular elements while the AF has limited involvement of these elements [21,25,26], not unlike some other connective tissues such as articular cartilage [27].

During development, the NP is mainly populated by cells of the notochord while cells of the AF are mesenchymal cells having a more fibroblast-like phenotype [28,29,30,31,32,33]. While the organization of the NP is more cartilage-like, the AF has a sophisticated structure [34] and the template laid down during development is initially cell-rich and matrix-poor, which then becomes cell-poor and matrix-rich during growth and maturation with a matrix being added according to the initial template, similar to the growth and maturation of other connective tissues as they mature from birth to skeletal maturity.

During growth and maturation and subsequent ageing, the NP appears to change [32], with notochordal cells dying and the matrix becoming more fibrocartilage-like. The age at which this happens is species dependent [35]. In contrast, this does not happen in the AF, but there can be an increasing risk for herniation of lumbar disc AF with protrusion of NP through a fissure in the AF or cartilage endplate leading to nerve impingement and sciatica. Also with ageing, degeneration of the AF can occur over time, leading to loss of integrity, loss of matrix organization and development of chronic pain [34]. For some of these conditions, the incidence appears to occur more frequently in females than males, possibly associated with pregnancy when increased loading and joint laxity is observed [36,37,38,39].

## 3. Immune Cells in Normal IVD Tissues

As the NP is neither innervated nor vascularized, any tissue-associated immune cells present are not there because of the direct vascular route. Furthermore, as the AF is minimally innervated and vascularized, it is unlikely that any tissue-associated immune system-related cells in the tissue are dispersed throughout the tissues via the vasculature as well. Details regarding when and how such cells get into the tissues or are maintained in the tissues remain to be elucidated.

One of the cells found in the normal human NP are mast cells [40], but this is controversial as other reports have indicated there are no mast cells in the normal NP or AF [41,42]. In the study by Wiet et al. [40], the level of mast cells was several folds higher in the NP than in the AF. Using immunolocalization techniques, such as immunostaining for mast cell tryptase, mast cells were readily detected in the NP but not the AF where levels were very low. As the studies by Peng et al. [41] also used an anti-mast cell tryptase, it is not clear why these authors did not detect mast cells in normal discs unless there may be ethnic differences. These authors did, however, detect higher levels of mast cells in degenerated human discs. Therefore, because of some of the nuances of detecting mast cells in normal IVD tissue, this issue should be revisited with additional research focus. Habtemariam et al. [42] also used anti-tryptase and anti-chymase approaches to assess the presence of mast cells in control IVD tissue from three individuals in Finland and failed to detect mast cells. Furthermore, if only anti-tryptase or anti-chymase are used to identify the presence of mast cells and the mast cells are degranulated, there could be a false negative result. Degranulated mast cells could still contribute to the local dysregulated environment via the release of mediators such as brain-derived neurotrophic factor (BDNF) [43,44]. Therefore, the basis for the inconsistency in detecting mast cells in normal control IVD tissues remains to be resolved in more detail.

While some confirmation is likely required, the reported distribution of mast cells in normal IVD tissue is of interest as in ligaments, mast cells have been detected and in direct association with neural elements in the epiligament [45] and in innervated fat deposits near the insertion of tendons into bone [46]. As there are no reported neural elements in the normal NP, the regulation of these mast cells in this tissue under normal circumstances must be somewhat different than these other tissues, possibly maintained in a non-degranulated state by the loading environment of the NP. Furthermore, the function of the mast cells in the NP also remains to be determined as they contain a large number of biologically active molecules, and thus their presence could put the NP or AF at risk if they became deregulated. Finally, it also remains to be elucidated whether the mast cells in the NP play a role in the age-related transformation of the tissue discussed above.

## 4. Mast Cells

Mast cells are bone marrow-derived cells that are most well known for their involvement in allergy and asthma [47]. Many mast cells circulate in the blood. However, mast cells, as components of the innate immune system, can also be associated with tissues and reside in connective tissues and thus may be heterogenous and consist of specific subpopulations [48,49]. Such mast cells can contain preformed granules that contain bioactive molecules [50] such as histamine and proteinases such as tryptase [51], chymase [52], or HTRA1 [53]. Some reports have suggested these proteinases could be drug targets [54,55] as some such as tryptase have been implicated in inflammatory processes [54,56].

Mast cells have been implicated as playing a role in aberrant skin wound healing in the red Duroc porcine strain but not normal skin wound healing in the Yorkshire strain pig model [57]. The formation of a hypercontracted skin scar in the red Duroc model was prevented by the mast cell stabilizer ketotifen [57]. Mast cells may also be involved in late wound healing events in mice [58], endometriosis and multiple sclerosis, as discussed in [59]. These cells have also been implicated in aberrant healing of joint injuries in both humans and rabbit models [60,61,62,63,64,65]. In a rabbit model of joint contracture development, treatment of the animals with ketotifen again alleviated ~50% of the contracture severity [62,63].

While mast cells have been implicated in several conditions (summarized in Table 1) and appear to be somewhat heterogeneous depending on their environment (i.e., tissue localized, circulating in the blood), their detection and quantification can be complicated by the methods that are employed in their detection, as reviewed in [66,67]. Thus, the detection of mast cells in tissues may employ cell staining using dyes or antibodies to find components such as tryptase or chymase, after appropriate fixation. Thus, if the mast cells become degranulated and release their proteinases, proteoglycans or other components that are central to the staining method, the cells may be erroneously classified as being absent (discussed in [68]). In such circumstances, specialized stains may be required to prevent coming to inappropriate conclusions, such as the use of pinacyanol erthrosine (discussed in [68]). This issue could account for the apparent lack of consistency regarding the presence of mast cells in normal IVD tissue that has been reported in the literature, i.e., [40,41].

## 5. Ageing, Injured or Degenerated IVD Tissues

Connective tissues of the IVD develop, grow and mature in a coordinated manner. In some respects, the IVD could be considered an organ system [74], similar to other joints such as the knee [14,15,16], but it likely should not be considered a diarthrodial joint [75]. Dysregulation of this IVD organ system can arise during growth and maturation with adolescent onset scoliosis as an example that occurs most often in females after puberty [76,77,78]. After maturity, the IVD can also exhibit changes due to what has been attributed to “ageing”. Whether this is due to use, overuse, genetics/epigenetics, or even life events such as pregnancy is not well defined or characterized. However, cellular and matrix changes to the NP appear to occur at defined timepoints in life in a sex-dependent manner [5,79], so some aspects of change to the IVD appear to be programmed. As with an organ system like the knee where loss of integrity of one component can lead to adaptation in other components or development of degenerative tissue such as osteoarthritis, as discussed in [80], age-related changes, injury, or impaired repair of elements of an IVD could lead to degeneration and loss of function.

### 5.1. Ageing

As humans age, the intervertebral discs undergo changes at both the biological [4,5,79,81,82] and biomechanical levels [83]. Both the annulus fibrosus and the nucleus pulposus can undergo changes but the changes in the NP are quite substantial as it goes from a more gelatinous tissue with the presence of many notochordal cells and a collagen II-rich matrix to one that is more fibrocartilaginous in nature. Interestingly, vigorous exercise may influence some aspects of this age-related process [81].

While the cellular composition of the NP changes with age [74,84], some of the notochordal cells are reported to be retained [85]. The matrix of the NP and AF changes with ageing as well [86,87,88,89]. These changes in the extracellular matrix of the IVD may result from genetic programming, microdamage and repair via use, epigenetic alterations, or indirectly via changes to the cell populations. Irrespective of the mechanisms, the alterations likely also contribute to changes to the biomechanics of the IVD tissues. Due to the complexities of the dynamics of the matrix and cells in the IVD tissues, it is often challenging to separate ageing and the initiation of degeneration of the tissue. Of note, information regarding changes in the mast cell content of the NP and AF of the clinically “non-degenerated” or ageing disc could not be found in the literature. Therefore, a possible role for deregulation of the endogenous mast cells to participate in the alterations to the IVD occurring during the ageing process may have some validity, but this possibility remains a speculation at this point in time.

Accompanying the ageing process in the IVD are alterations to the biomechanical properties of the tissues [83,90]. While the NP alterations leading to a transitioning from a very proteoglycan-rich tissue to a more fibrocartilage-like tissue would lead to altered biomechanics, there are also changes to the AF as well with ageing [91], including changes to the interlamellar biomechanical properties [92].

The mechanistic basis for the age-related loss of NP integrity is not well understood [4,5]. One option is that it is due to increasing mechanical loading; the endogenous cells making the ECM become overloaded and undergo some version of cell death or transformation to make a more fibrocartilage-like matrix to withstand the loading. Another option is that the endogenous mast cells become dysregulated enough to impact the endogenous cells in a manner that is insufficient for initiating a systemic inflammatory response. The controlled degranulation of the mast cells would potentially lead to an amplification of the process to impact a large number of endogenous cells/mast cells. As such, the tissues would remain isolated and not subjected to systemic influences, which may be detrimental. Whether the mast cells had become dysregulated in a controlled manner via the biomechanical environment or due in part to endocrine factors penetrating the tissues via diffusion remains to be determined. If this latter speculation is confirmed by future investigation, then it could lead to better understanding of why the mast cells are present in the NP, and to a lesser extent in the AF, as part of the regulatory apparatus of the IVD across the lifespan.

**Figure 1:**
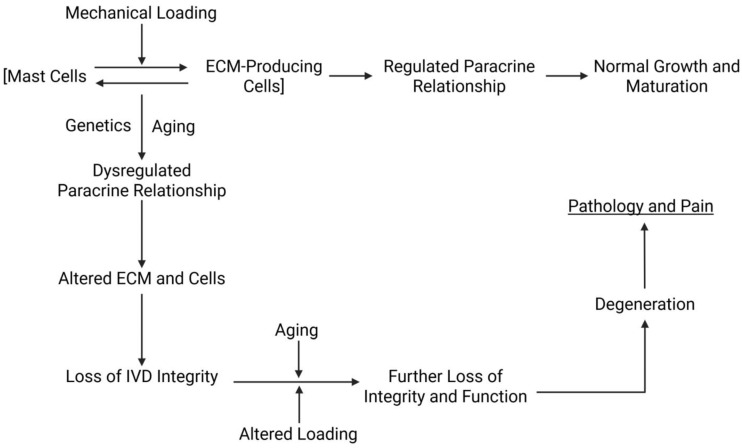
Possible paracrine relationship between endogenous mast cells and notochordal cells in the regulation of NP integrity in health and disease development. In the dynamic loading environment of the NP, mast cells and ECM-producing cells such as notochordal cells function in a paracrine relationship with factors released by one affecting the other and vice versa. This relationship regulates normal growth and maturation and may become dysregulated during NP transitions with development of pathology.

An alternative option is that the mast cells and endogenous cells making the NP ECM are functioning as a paracrine unit, with the mast cells functioning in a manner outside their somewhat traditional pro-inflammatory or pro-allergy role. Previously, a model was proposed that suggested there was an axis consisting of neural–mast cell–myofibroblasts operative in wound repair. As there are no neural elements in the normal NP, there could still be a bidirectional paracrine “axis” where the endogenous cells such as notochordal cells secrete factors that impact mast cells, resulting in “regulated” degranulation of the secretion of factors that then affect the metabolism of the endogenous cells (Figure 1). The precedent for such a relationship is supported by the report of Weit et al. [40], who reported that conditioned medium from notochordal cells could affect mast cells and vice versa. While Weit et al. [40] were focused on degenerated IVD, such interactions could also pertain to a relationship in non-degenerated tissues. All of this activity would be occurring in the intermittent compression mechanical environment. Alterations to the mechanical environment, such as during ageing, may induce dysregulation of this paracrine relationship contributing to the age-dependent decline in the integrity of the IVD.

Weit et al. [40] also reported that human AF contained fewer mast cells than did the NP. From the data presented, it was not clear as to whether the mast cells in the AF were widely distributed or were associated with particular features of the tissue such as the interlamellar matrix. Irrespective of location, mast cells may serve a function similar to that proposed for the NP, but it may be somewhat different as the mechanical environment in the AF differs from that of the NP.

Some questions regarding “normal ageing” of the NP and AF remain to be answered by further research. First, do all discs of an individual age at the same rate, possibly indicating a role for genetic factors influencing biological ageing? If so, are some individuals at higher risk to develop more rapid ageing, which may put them at risk to develop clinically relevant pathology? If true, then it may be possible to identify the genetic features that pose such risk. Genetics have been reported to play a role in disc degeneration [6,7,8], but whether genetics also relates to “normal ageing” remains to be elucidated. It also remains to be determined if some individuals are genetically resistant to “normal ageing” and also resistant to overt clinical degeneration. Second, if discs age asynchronously, is there a pattern that may depend on the biomechanical environment? If no pattern exists, what is considered “normal ageing” may be influenced by microtrauma or other life events.

Several life events may uniquely impact the ageing of IVD components differently in females compared to males. After puberty, the onset of adolescent idiopathic scoliosis occurs primarily in a subset of females [76,93,94], indicating hormones such as estrogens play a role in IVD growth and maturation [95]. Many females experience lower back pain and involvement of lumbar discs when pregnant [96], possibly due in part to increased laxity. Furthermore, loss of systemic hormones due to menopause is also reported to cause alterations to IVD integrity (discussed in [97,98]). Thus, these life events in females may contribute to the rate of “normal ageing” of IVD, as well as contribute to the development of clinically relevant pathology.

### 5.2. Sciatica/Disc Herniation

Lower back pain can arise from herniation of the IVD, leading to sciatica (reviewed in [99,100,101]). These herniations usually arise in the lumbar region and in the L4/5 and/or L5/S1 discs. In most cases, NP material protrudes from the herniated AF, but in some cases, pain can arise from the bulging of the weakened AF material without a complete rupture of the AF [102]. This process leading to AF herniation is reported to be associated with fibrotic alterations to the annulus tissue [103].

The incidence of sciatica in males versus females is ~2/1 and a frequent age bracket for development is 30–50 years of age. Interestingly, with treatment of the pain with medications over ~2–4 months after the onset of sciatica (confirmed by MRI), the condition resolves “spontaneously” in ~75–85% of cases with endogenous resorption of the extruded NP material and apparent sealing of the herniation [104,105,106,107,108]. However, the pain persists in 15–20% of patients with an apparent failure to resorb the extruded NP material. For those with persistent pain (~15–20% of patients), surgical treatment is often undertaken (reviewed in [109,110,111]).

While some modalities such as MRI have been used to diagnose sciatica or lumbar disc prolapse [112], other reports have attempted to analyze blood from patients with or without the condition to determine whether systemic changes can be detected in patients. In a very small study (8 patients—4 male and 4 female) with controls that were much younger than the patients, Wang et al. [113] reported that the transcriptome signature of the patients was different from that of the controls and revealed a number of candidate genes that may be biomarkers. More recently, further studies have reported the identification of immune-related biomarkers in the peripheral blood of patients with sciatica compared to controls [114,115]. Many of the targets identified were related to inflammation, an area that might be expected given the intense pain and reaction to the condition. An additional study used cerebrospinal fluid (CSF) from 10 patients and 10 controls and 2D electrophoresis to detect differences between the two populations [116]. These authors detected a number of differences including a deficiency of cystatin C in the CSF from the patients. While the focus of these studies was to detect differences between patients and controls without sciatica, studies whose goal was to detect differences between sciatica patients whose condition resolved versus did not resolve over time without surgery have not been reported. Therefore, the mechanism(s) responsible for these differences have yet to be understood.

However, what is somewhat amazing is that the disc can undergo herniation, which can subsequently and effectively repair itself in a high percentage of cases, and without catastrophic collapse of the function of the disc. Thus, even with herniation of the IVD structure, the complex structure of the matrix retains significant integrity and allows for the herniation to resorb and re-establish integrity in a high percentage of cases. Thus, the matrix of the AF and the endplate are well designed and very resilient to failure after the development of the localized defect.

### 5.3. Degenerated IVD Tissues

In contrast to sciatica, which presents as an acute condition with severe pain, disc degeneration likely occurs over a long period of time, presenting as chronic pain with often increasing severity that does not resolve [117]. As the intervertebral disc changes with ageing (discussed above), overt degeneration and ageing changes can proceed in tandem and segregating their relationship can be challenging. Degeneration can progress in different manners (i.e., with and without deformities), which further complicates understanding the commonalities and differences that progress to different phenotypes. Whether some of the conditions associated with lower back are associated with bipedal walking or other variables is not clear. However, some degeneration phenotypes exhibit sex differences in humans [5,118] and in mice [119]. Some evidence from mouse models [119] indicates that genetic factors are involved and this has also been implicated in some human studies [120] but not in others [121]. Of note, many women develop low back pain during pregnancy that may or may not resolve or may become more severe after multiple pregnancies, which leads to the conclusion that pregnancy-associated increases in spine laxity and/or the additional stress of carrying a fetus may be a contributing factor for developing disc degeneration later in life. This may also be associated with lumbar disc degeneration of various extents based on MRI assessments [122]. However, some reports do not support a progression in disc degeneration with an increasing number of pregnancies [123].

As disc degeneration likely has a lengthy time course prior to becoming symptomatic, not unlike osteoarthritis or the hip or knee, it is nearly impossible to deduce the initiating events or processes that contribute to the onset of overt IVD degeneration, although perhaps the validation of blood biomarkers may assist in resolving this issue [124]. Most samples obtained at the time of surgery are by necessity from patients with fairly advanced disease, or at a minimum, with severe symptoms of pain and disability. Therefore, nearly all human studies are by necessity cross-sectional in nature, a limitation that compromises some experimental approaches. In contrast, control or non-degenerated tissue can often be obtained from tissue procurement centres at the time of autopsy or from banked tissues that have been frozen and/or fixed in some manner.

In the AF tissue of degenerated human discs, collagen integrity is compromised [125,126,127], as well as that of proteoglycans [128,129,130]. The expression of a number of other molecules is also altered in degenerated human discs [113,131,132], particularly the AF [133]. These include serglycin [134], MMP-28 [135], CK8 [136], asporin, a small leucine-rich PG derived from fibrillin-1 [137], connective tissue growth factor [138], and Interleukin-17 [139]. Many of these changes appear to be related to the fibrotic-like alterations to the different disc tissues occurring during the degeneration process [140,141,142]. Some of these molecules can be detected in plasma and could serve as biomarkers of disease and disease progression [143].

In degenerated disc tissue there can be an infiltration of vascular and neural elements into the normally avascular and aneural tissues, as discussed in [144,145]. Based on such infiltration, some signals must be generated within the tissues during the degeneration process, which allows for such chemoattraction changes, possibly from endogenous cells such as mast cells or those generating ECM molecules. As a consequence of the changes, inflammatory and other cells of the immune system can be introduced into the tissues and the neural elements could contribute to the pain associated with the degeneration or the release of neurotransmitters such as Substance P or CGRP, which could affect disc tissue and cells [146,147,148,149,150] or cells of the immune system such as mast cells [151,152,153] and non-immune resident connective tissue cells [154,155,156]. Of note, in some connective tissues such as tendons and ligaments of preclinical models, pregnancy impacted the effect of SP and CGRP on the tissues [157,158], likely indicating regulation of tissues by neuropeptides in males and females may be different [158] and that sex hormones may play a role in tissue responsiveness to neuropeptides. This should be further explored using intervertebral disc tissues, particularly since expression of proteinases such as MMP-3 were affected by neuropeptides in human tendon cells [156] and pregnancy in preclinical models [157]. Thus, there may also be a neural–mast cell interaction axis in the regulation of cell function in the diseased IVD.

The altered cellular and ECM environment in the degenerating disc [159,160,161] leads to alteration in the structural integrity of the matrix of the disc [162,163,164,165]. This can lead to the loss of the lamellar structure of the AF (altered number and function of lamella or complete loss of lamella) [126,138] and loss of the NP tissue as detected by MRI using the Pfirrmann Scale discussed in [166,167,168]. The loss of the integrity of the lamellar structure of the AF and the fibrosis of the tissue can contribute to the decline of the biomechanical properties of the tissue [125,126,169]. However, as mentioned earlier, the assessment of disc tissue obtained at the time of surgery likely reflects tissue from patients with advanced disease, and thus, does not reflect the early inductive stages of the conditions.

In summary, there are a number of biological changes occurring in the degenerated IVD, as well as mechanical changes. One issue that remains to be resolved is whether changes to the mechanical environment led to the biological changes or vice versa. As discussed in Section 5.1 on ageing, during the ageing process one can detect changes in the structural integrity of the IVD as well as biologic changes without the appearance of symptoms. Whether subsequent changes to the mechanical environment lead to further alterations to the biological environment or further biological changes and loss of ECM integrity lead to an inability to maintain mechanical stability remains to be resolved. However, once initiated as DDD, the mechanical environment and an inflammatory environment may work together to contribute to process of the IVD degeneration [170]. This may also initiate a fibrotic response in an attempt to repair the affected tissue (reviewed in [171]).

## 6. Rationale for Using Mast Cell Stabilizers in IVD Conditions

As mast cells have been detected in tissues of the IVD and their numbers appear to vary with degeneration [40,41], it is possible that they are involved in both normal processes in the disc and their dysregulation plays a role in the degeneration process possibly via the release of bioactive molecules such as proteinases and mediators. This hypothesis was raised by Freemont et al. [68] who also proposed that mast cells could be an attractive therapeutic target. This concept was further supported by He et al. [172]. Thus, inhibition of degranulation could elucidate some aspects of their involvement in IVD disease.

### 6.1. Previous Use of Ketotifen and Sodium Cromoglycate in MSK Diseases or Following Tissue Injury

While mast cells are usually thought of in the context of allergies and asthma, the various subsets of mast cells (i.e., tissue mast cells, mucosal mast cells, circulating mast cells) can play central roles in a variety of processes ranging from abnormal skin wound healing [57], development of both human joint contractions [60,61] and joint contractures in a preclinical model [62,63,64,65,173], human patellar tendinosis [72], mouse patellar tendon repair [174], flexor tendon repair in a rabbit model [175], and postulated to play a role in multiple sclerosis and endometriosis (discussed in [59]) to name a few examples (summarized in Table 1). In human elbow contractures, the elevated mast cell numbers in the contracture are accompanied by elevated levels of mast cell tryptase in the blood [176]. Elevated levels of tryptase were also detected in the serum of rabbits with an experimental knee joint contracture [177].

Drugs such as ketotifen and sodium cromoglycate are molecules that have an extensive use profile, are safe even for pediatric use, are off patent protection, and stabilize mast cells to inhibit or prevent degranulation (discussed in [57,61,65,173]). Thus, these drugs are well tolerated and effective, with a long track record of safety.

In the red Duroc pig model of hypercontracted and hyperpigmented full thick dorsal skin scars, oral administration of ketotifen led to a prevention of the development of the abnormal healing phenotype if given early after skin injury [57]. If given later after injury, the treatment was ineffective, indicating that the drug could not reverse the impact of early events. Interestingly, if given early after skin wounding in the Yorkshire pig model, a breed that heals normally, the drug was without effect [57], indicating that it was effective only when the healing phenotype was abnormal. Thus, mast cells do not appear to be central to normal skin wound healing but appear to play an important role in abnormal skin wound healing, a role that can be blocked by early oral ketotifen treatment. However, wound healing is an acute event, and the role of mast cells is restricted to early events, so effectiveness in chronic conditions is still unknown.

In a rabbit model of joint contracture, a knee joint injury plus immobilization of the joint leads to loss of joint motion and development of a joint contracture [63,64]. Treatment of the rabbits with subcutaneous ketotifen diminished the extent of the contractions by ~50%. Ketotifen treatment also diminished the mast cell tryptase levels in the serum of rabbits with contractures [176,177]. Currently, there is an ongoing clinical trial of ketotifen in human elbow contracture development [178,179], and efficacy of the intervention could be known in the near future. Again, details regarding the timing of when such an intervention is provided, post injury or post-surgery, may influence the outcome of the intervention.

Based on the examples discussed above (summarized in Table 2), the extensive safety profile of the drugs, and the reports of potential mast cell involvement in IVD diseases and conditions, clinical trials of ketotifen and sodium cromoglycate use in human sciatica and DDD may be warranted. The selection of these two conditions is based in part on the reported presence of mast cells in the tissues and the fact that sciatica is an acute disease while DDD is a chronic disease that likely has a very long disease progression after some inciting event and perhaps an inherent risk involving genetic or epigenetic variables.

**Table 2:** Use of mast cell stabilizers in connective tissue repair.

Mast Cell Stabilizers	Species	Condition	Effective (Y/N)	Ref.
Ketotifen	Yorkshire ^1^ Pig	Skin Wound Healing	N	[57]
Ketotifen	Red Duroc ^2^ Pig	Skin Wound Healing	Y	[57]
		Early After Injury ^3^	Y	[57]
		Late After Injury ^4^	N	[57]
Ketotifen	Rabbit	Knee Contraction	Y	[62,63]
Sodium Cromoglycate	Mouse	Patellar Tendon Healing ^5^	Y	[174]
Ketotifen	Human	Post-Traumatic Elbow Contractures	? ^6^	[178,179]

^1^ Mast cells present but skin wounds heal normally. Normal healing not affected by oral drug. ^2^ Skin wounds heal with an abnormal hypercontracted, hyperpigmented phenotype. ^3^ Oral ketotifen started at the time of injury. Alleviated abnormal phenotype. ^4^ Oral ketotifen started at day 28 after injury. No effect on phenotype. ^5^ Drug-inhibited fibrotic response during wound healing. ^6^ Multicentre clinical trial ongoing with oral ketotifen.

While mast cell stabilizers are safe, the ability to deliver the drugs to tissues that are hypovascularized could be a challenge. Normal NP is avascular and aneural so it is somewhat sequestered from the circulation. Delivery of drugs to such a sequestered environment of the normal IVT may require some preliminary studies in a large animal such as the domestic pig, which has a physiology similar to humans to assess whether the drug actually can get to the NP and AF. As pigs have been used in previous studies [57], drug delivery via the oral route should not pose significant challenges. As degenerated human IVD undergoes neovascularization (discussed in Section 5.3), the delivery of drugs to this altered environment may not be an issue.

### 6.2. Potential of Use of Ketotifen and/or Sodium Cromoglycate in Sciatica

Sciatica with lower back pain that can radiate down the leg often has a rapid onset and is often accompanied by a herniated disc with protrusion of NP tissue via a fissure through the AF as detected by imaging, usually via MRI [112,180]. Initial treatments are usually the use of pain medication, exercise/physiotherapy and rest as approximately 80–85% of cases resolve on their own after 3–4 months while in 15–20% of cases the pain persists, and the patients often require surgery. Why this subset fails to resolve is not well understood.

As the NP is reported to contain more mast cells than the AF [39], the protrusion of the NP into a new environment could lead to the cells becoming activated and degranulated. As it is known that degranulated and activated mast cells can release a variety of biologically activated molecules, these could contribute to the outcomes. Mast cells are known to express a brain-derived neurotrophic factor (BDNF), a molecule that could contribute to alterations in neural elements [43,44]. Mast cells also can release proteinases such as tryptase, which is pro-inflammatory [51,181], and HTRA-1 [53], which could cleave matrix molecules and help degrade the protruding NP tissue leading to resolution of the pain. Alternatively, as mast cells can play a role in fibrosis (discussed in [62,63,64,65]), activation of mast cells in a different manner could potentially lead to conversion of the protruding NP into a fibrotic mass that perpetuates the pain until the aberrant tissue is removed via surgery.

**Figure 2:**
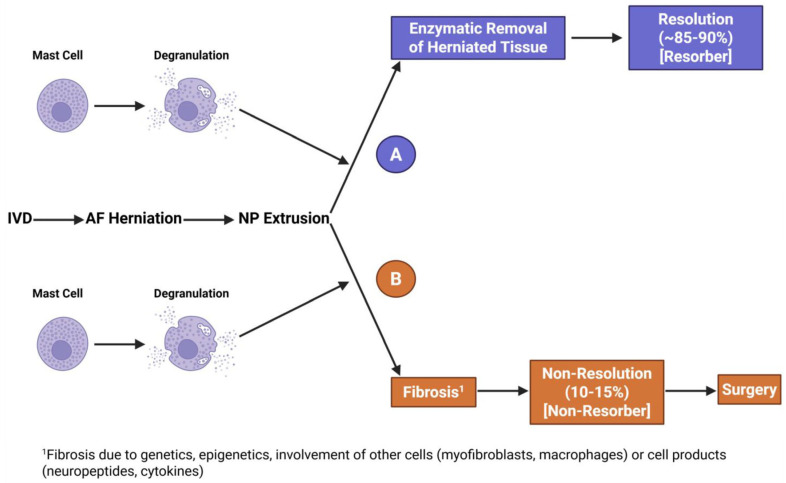
Potential involvement of mast cells in outcomes after development of sciatica. There are two options for the involvement of mast cells, immune cells known to be in normal NP. **Option A**: The mast cells in the extruded NP degranulate leading to the release of proteinases and other molecules that degrade the NP tissue, which eliminates the pressure on nerves causing pain. Patients undergoing this sequence would be labelled “resorbers” and not require surgery. **Option B**: The mast cells degranulate but the process leads to an activation of endogenous or infiltrating myofibroblasts, leading to a deposition of collagen and a fibrotic outcome. In this case, the extruded tissue would not be resolved, pain would continue, and patients would require surgery. These patients would be labelled “non-resorbers”. Patients progressing via Option A versus Option B may be influenced by genetics, epigenetics, or other factors. Created with BioRender.

Thus, mast cells could be central to both resolution of sciatica in the majority of patients if their products contributed to resorption of the protruding tissue or the perpetuation of the problem if in a minority of the patients the mast cell products contributed to fibrosis of the protruding tissue and a failure to resorb (Figure 2, Option A vs. Option B). Thus, it may be risky to treat all sciatica patients with mast cell-targeting drugs early in the disease process as they could have a negative impact on outcomes for the majority and a positive impact on a minority of patients. What is likely needed before pursuing this line of investigation is the development and validation of biomarkers, obtained from imaging modalities or perhaps via identification of biomarkers in the blood of patients whose condition resolved versus those who do not, and thus, the subset having a non-resolving phenotype could be targeted specifically.

As an initial assessment, one could assess for the presence of mast cell tryptase in serum from patients that ultimately resolve or do not resolve symptoms and the herniation to confirm that mast cells may be involved in the process impacting outcome. This has been done in the past for both preclinical models in a rabbit [177] and in patient populations [176]. The initial comparisons for the presence of mast cell tryptase would be between serum from patients with sciatica taken at time 0 (onset of symptoms) and then 4, 8, 12, and 16 weeks post-onset and serum from age-matched males and females without sciatica. For the females, care would be needed to control for the phase of the menstrual cycle if the females had not reached menopause as changes in estrogen/progesterone levels could influence mast cell degranulation [182,183]. Once it is established that serum tryptase levels are elevated during the course of sciatica, one could evaluate levels in those that resolve naturally versus those that do not resolve and ultimately require surgery.

Depending on the results obtained with the above discussed approach, additional biomarker identification could potentially be elucidated using techniques such as proteomic analysis [184,185] on serum or plasma taken from a panel of patients during their disease course such as at the onset of symptoms and then at intervals post-symptom onset, with recordings of resolution versus non-resolution of symptoms and imaging validation of loss or persistence of disc herniation tissue. This approach using methodologic variations in mass spectrometry can be a powerful tool to identify biomarkers in complex mixtures and assess their interrelationships regarding pathways and networks of molecular events that are predictive of outcomes [56,186,187]; reviewed in [188].

Thus, the potential use of mast cell stabilizers such as ketotifen or sodium cromoglycate in sciatica may have validity, but before entertaining clinical trials, a body of research will likely have to be performed to assess potential risks to the patients. This may include development of a preclinical model of acute herniation of the AF in a relevant species to conduct preliminary studies.

One very good choice for such a preclinical model is the domestic pig. This large animal has a physiology similar to humans, is available in several different breeds that differ genetically and are only ~20% inbred and is a species that has been used previously in IVD research [189,190,191,192,193]. These studies used domestic pig breeds or minipigs to study the IVD. However, no studies could be found that utilized the red Duroc breed that heals skin [194] and knee ligament injuries [195] with altered phenotypes compared to the Yorkshire breed, and in the case of skin wounds, is amenable to interventions with ketotifen [57]. Such studies in red Duroc pigs are also amenable to genetic studies to assess inheritance patterns of the healing phenotypes [196,197,198].

Groups of Yorkshire and red Duroc animals could be bled for serum and then a stab wound of the AF of specific IVD could be used to induce extrusion of NP and development of a sciatica-like condition. The resolution of the extruded tissue (resorption) or failure to do so (non-resorption/fibrosis) can be monitored via assessment of animals at different time points post-injury and biomarker analysis of serum obtained at different time points post-injury. Based on outcomes, further studies using ketotifen could be envisioned to assess the role of mast cells to impact outcome differences between breeds. Such studies could then be used to inform future clinical trials, with proteomic analysis of pre- and post-injury serum samples used to identify potential biomarkers of outcomes.

Integration of the preclinical model outcomes with the characterization of the histologic and molecular analysis of human samples of non-resolving sciatica tissue obtained at surgery could provide direction to developing clinical trials regarding mast cell involvement in outcomes in patient populations.

### 6.3. Potential Use of Ketotifen and Sodium Cromoglycate in IVD Degeneration Conditions

In contrast to the acute nature of sciatica, development of DDD likely may take years or decades before symptoms become so severe that surgery is required. DDD is actually an umbrella term as there are subsets of patients based on molecular features of the conditions [199,200,201]. Either these phenotypes arise from different consequences to the initial insults leading to DDD or there is a common mechanism involved that arises due to the initial loss of integrity and development of symptoms, usually associated with pain. Thus, while labelled DDD, it is also not known in detail whether the progressive loss of integrity of the IVD is due only to degeneration or whether there is also a risk for the development of symptoms due to attempts to repair the initial disruption of integrity. One possibility is that mast cells and their products are involved in either the degradative process or in the attempted repair process via fibrosis-inducing mechanisms [68], possibly by stimulating inflammation and fibrosis.

Mast cell activation has been detected in tissues from surgical patients with DDD [202] and it has been proposed that mast cell–intervertebral disc cell interactions are involved in discogenic back pain [40]. There have also been reports of mast cell tryptase in tissues from patients (discussed in [203]) and this proteinase can be considered an inflammatory mediator [181] and an angiogenic factor [51]. In addition to the ability to degrade extracellular matrix molecules, tryptase can activate proteinase activated receptor 2 (PAR2) and contribute to induction of inflammatory processes [71,193]. Furthermore, HTRA-1, a proteinase also expressed by mast cells [53], has been implicated in DDD [141,204,205,206], particularly in female Japanese patients [207]. HTRA-1 may degrade chondroadherin during disc degeneration [204] and may be a marker for disc cells [208]. HTRA-1 may also affect disc degeneration via induction of MMP-3 [209], another proteinase implicated in disc degeneration [210]. Interestingly, both HTRA-1 and MMP-3 can be induced in intervertebral disc cells by activation of toll-like receptors, leading to increased extracellular matrix degradation in the tissue [211]. Relevant to this point are findings indicating that PRG4 can interact with toll-like receptors [212,213] and potentially influence inflammatory processes.

Additionally, AF has been reported to have much more PRG4 than NP and this increases with age [214], similar to what has been reported for ligaments [215]. The loss of this lubricating molecule appears to contribute to altered mechanical functioning of the AF [216], similar to what is known about its role in other connective tissues such as tendons [217]. How and why PRG4 increases in the AF with age is not known, and what the consequences are regarding function is also not well understood. However, as different connective tissues are reported to contain different splice variants of PRG4 [218], there may be tissue-specific functions of this molecule. Furthermore, cleavage of PRG4 by mast cell tryptase has been reported to contribute to inflammatory processes in the knee [56]. Thus, there could be an interplay between PRG4 and mast cells in IVD homeostasis, ageing and development of DDD or disc herniations. However, given the evidence for involvement of a spectrum of serine proteinases (i.e., HTRA1) and MMPs (i.e., MMP-3 and others) in both IVD degenerative diseases, the issue arises as to whether interventions using mast cell stabilizers alone should be the focus, or whether a “cocktail” of specific proteinase inhibitors and mast cell stabilizers should be entertained.

In spite of the above concern, an initial step in improving understanding of the role of mast cells in DDD could be the assessment of mast cell tryptase levels in serum from patients, similar to what has been done in patients with elbow injuries [176] and in rabbits with joint contractures [163,168]. This assessment could be performed with patients symptomatic with pain and DDD confirmed by imaging, as well in a prospective manner in at risk populations with back pain but are non-atopic and without DDD as determined by imaging. As females are reported to experience DDD at a higher incidence than males, and many women develop low back pain during pregnancy, with some having the pain persist post-partum and risk for development of DDD, this population may be appropriate to assess for serum tryptase levels in a longitudinal manner to determine whether activation of mast cells is occurring long before the DDD is evident by imaging. Such assessment of mast cell tryptase could also be used to determine whether there may be mechanistically separate subsets of patients who develop DDD. Such information could be used to develop more precision medicine approaches to treatment early in the DDD process, a stage when effective interventions would potentially have more impact on the retention of IVD integrity. Further validation of this approach using assessment of the proteinase HTRA-1 could also be performed as this proteinase has been implicated in DDD, particularly females [207]. While this proteinase can be expressed by mast cells [53], other cells can express it as well [219] so it may not be as specific a biomarker as is the mast cell tryptase. A third proteinase that has been implicated in DDD is MMP-3, a proteoglycanase formerly known as stromelysin [210,220]. Elevated levels of MMP3 in both disc tissues and plasma have been noted. Thus, proteinase regulation and mast cells appear to be interrelated in IVD disease, supporting the concept of an intervention containing both proteinase inhibitors and mast cell stabilizers.

Once it is established that serum tryptase levels are elevated in at least some DDD or pre-DDD patients, this information could also be used to monitor the effectiveness of treatment interventions. This has been successfully achieved previously in a rabbit model of joint contractures [168]. Similar approaches can be used for HTRA1 and MMP3, or the use of proteomic methods could be employed.

Before generating the “cocktail” approach to intervention, following a positive finding for tryptase, HTRA1 and MMP3 in the plasma/serum of patients with DDD use specific proteinase inhibitors to evaluate their effectiveness in alleviating symptoms of DDD and inhibiting disease progression could be entertained. While feasible, this approach does not have a promising track record, and the proteinases being targeted may also serve important normal functions, which could lead to side effects of proteinase inhibitors that limit their use. A more promising approach may be to target the cells that are involved and ones that can release multiple potential targets to create an inflammatory and/or catabolic environment in the IVD.

Using effective doses of ketotifen or sodium cromoglycate, both mast cell stabilizers of degranulation in DDD remain a viable option to test for effectiveness. While administering them late in the disease process may not lead to a reversal of damage to the IVD, the drugs could potentially still diminish continued damage by inhibiting inflammation. Relevant to this point are the findings of Peng et al. [41], who detected mast cells in the NP of patients with advanced DDD. Thus, given the safety profile of these drugs, pilot studies could be undertaken to evaluate effectiveness related to symptoms such as pain and mobility. However, the fact that mast cells could contribute to DDD progression and sciatica outcomes in ways other than just degranulation of molecules such as tryptase, such as chronic secretion of BDNF and cytokines, should not be forgotten as this could influence the interpretation of effectiveness or lack of effectiveness.

While the stabilization of mast cells has potential, the reality of any intervention is that mast cell involvement in DDD or sciatica may only be part of the story and thus, mast cell stabilization is not going to be a “silver bullet” and control all aspects of DDD and sciatica. Previous studies of ketotifen [57,62,63] indicated that effectiveness was only evident when it was started very early after tissue injury, so this may also be a concern regarding use in DDD. However, Peng et al. [41] reported elevated numbers of mast cells in tissue from patients with advanced DDD, so mast cell stabilization may be very effective. In this regard, the assessment of serum for tryptase, HTRA-1 and MMP-3 may lead to a change from early disease to later stages of disease and thus may provide a continuous profile of disease progression with stage-specific multi-modal interventions needed as the disc degeneration becomes more evident and progressive.

## 7. Involvement of Other Immune System Cells in Sciatica and DDD

While the focus of this article has been on mast cells, it is also clear that other cells of the immune system may also play roles in sciatica resolution and progression of DDD. However, evidence for endogenous immune cells other than mast cells in the normal AF and NP, particularly for human tissues, could not be found. Therefore, much of the literature has focused on the role of macrophages and lymphocytes in the response to disc herniation/sciatica and overt DDD rather than normal tissues and their ageing. Thus, the role of other immune cells such as macrophages and lymphocytes in disease initiation versus resolution or progression via induction of an inflammatory environment may be different in some respects from endogenous mast cells. That is, disease induction in DDD or the initial response in disc herniation/sciatica are different, and thus the cells may be different once a response is initiated.

NP is often considered an immune privileged tissue as it is aneural and avascular and thus sequestered during development and maturation (discussed in [221,222]). Exposure of the herniated NP tissue to an abnormal environment could then lead to attraction of immune cells in the circulation via a response to chemotactic factors released from the tissue leading to infiltration of antigen-specific lymphocytes and other cells such as macrophages [223,224,225,226]. In a mouse model, Haro et al. [227] provided evidence that macrophages attracted to herniated NP could release VEGF that promotes neovascularization and enhanced disc resorption. Several aspects of how macrophages and metabolic alterations impact the process of resorption were recently reviewed by Chai et al. [228] and Gong et al. [229]. With regard to macrophages, outcomes and the inflammatory environment may also pertain to the roles of M1 and M2 macrophages, which likely contribute to the inflammatory environment and resolution of the herniation differently (reviewed in [229,230]).

As discussed earlier, development and progression of DDD is a chronic disease, likely taking years to develop before becoming symptomatic, and is accompanied by innervation and neovascularization. Thus, cells of the immune system could gain access to the NP via the neovascularization. In addition, the AF can undergo loss of structural and mechanical integrity during DDD [125,126], again with evident neovascularization and areas of hypercellularity. Immune cells other than mast cells also contribute to the characteristics of DDD (reviewed in [231]). One of these cells is the macrophage [232,233] with macrophage polarization and inflammatory cytokines playing specific roles in the process [221,234,235,236,237]. In addition, once-activated macrophages can release several proteinases including MMP-13, which has been implicated in IVD degeneration (discussed in [238]).

In summary, endogenous mast cells may play an important role in the initiation of host responses to herniated NP tissue or in DDD, but other immune cells, particularly macrophages and perhaps T-lymphocytes also play important roles in contributing to the outcomes following disc herniation and in DDD. As disc herniation/sciatica is an acute event and DDD is likely the result of a chronic process, understanding how endogenous mast cells and other cells of the immune system interact requires much additional research. Research on early events in the processes leading to human conditions is particularly challenging as often tissue is obtained at the time of surgery when the DDD is well established, and the environment is near the end stage. Similarly, most often tissues are obtained at surgery when the resorption or non-resorption processes are well underway.

## 8. Conclusions

Several reports have indicated that mast cells are present in IVD tissues, with many more in the NP than the AF [40]. Based on their presence in IVD tissues and changes observed in tissues from patients with DDD, it has been hypothesized that mast cells play a critical role in DDD progression, and possibly initiation [202,203]. While an intriguing possibility, data to support a central role for mast cells in such chronic conditions are still incomplete. However, the use of mast cell stabilizers in other connective tissue conditions such as abnormal skin wound healing [57] and knee joint contracture development [62,63,173] support the perspective that their use in treating DDD development and progression should be investigated. Furthermore, the possible role of mast cells in the natural resolution/non-resolution in acute sciatica should be further investigated given the levels of mast cells in the NP [40] that finds itself in an abnormal environment in the herniated disc. While application of mast cell stabilizers in this condition may require more preliminary research as in this condition, mast cells may play differing roles in those that resolve their sciatica versus those who do not. Appropriate application could potentially lead to risks that need to be more completely understood. As drugs with good safety and efficacy records for other conditions are available for use in patient populations, pilot studies that can be readily undertaken to assess the efficacy of mast cell stabilizers in DDD and sciatica to provide a non-surgical option to improve clinical outcomes and increase understanding of the role of mast cells in the conditions are warranted. These studies should also be integrated with the study of other immune system cells to provide a more complete analysis of the cellular spectrum contributing to the outcomes.

## Figures and Tables

**Table 1:** Observed and postulated roles of mast cells in connective tissues and joints.

Species	Tissue	Observed and Potential Role(s)	Refs.
Human	Skin	Allergic reactions	[69]
			[70]
Pig	Skin	No detectable role in normal healing	[57]
		Abnormal healing—hypercontracted scar	[57]
		Neuroinflammatory axis	[64,65]
Rabbit	Knee Joint	Joint contracture development	[62,63]
		Neuroinflammatory axis	[64,65]
Human	IVD-AF	Contributes to degenerative process	[40]
	IVD-NP	Contributes to tissue degeneration	[40]
Canine	IVD-NP	Pro-inflammatory processes in tissue degeneration	[71]
Human	Patellar Tendon	Tendinopathy?	[72]
Rabbit	MCL ^a^-epiligament	Neuroregulation?	[45]
Human	MCL ^a^-epiligament	Unknown	[73]
Rat	Achilles Tendon	Neuroregulation/Neuroinflammation	[46]
	Enthesis		

^a^ MCL = Medial collateral ligament of the knee.

## Data Availability

No new data were created or analyzed in this study. Data sharing is not applicable.
